# Glyoxalases in Urological Malignancies

**DOI:** 10.3390/ijms19020415

**Published:** 2018-01-31

**Authors:** Cinzia Antognelli, Vincenzo Nicola Talesa

**Affiliations:** Department of Experimental Medicine, University of Perugia, 06132 Perugia, Italy; vincenzo.talesa@unipg.it

**Keywords:** Glyoxalases, urological malignancies, prostate cancer, kidney cancer, bladder cancer testicular cancer

## Abstract

Urological cancers include a spectrum of malignancies affecting organs of the reproductive and/or urinary systems, such as prostate, kidney, bladder, and testis. Despite improved primary prevention, detection and treatment, urological cancers are still characterized by an increasing incidence and mortality worldwide. While advances have been made towards understanding the molecular bases of these diseases, a complete understanding of the pathological mechanisms remains an unmet research goal that is essential for defining safer pharmacological therapies and prognostic factors, especially for the metastatic stage of these malignancies for which no effective therapies are currently being used. Glyoxalases, consisting of glyoxalase 1 (Glo1) and glyoxalase 2 (Glo2), are enzymes that catalyze the glutathione-dependent metabolism of cytotoxic methylglyoxal (MG), thus protecting against cellular damage and apoptosis. They are generally overexpressed in numerous cancers as a survival strategy by providing a safeguard through enhancement of MG detoxification. Increasing evidence suggests that glyoxalases, especially Glo1, play an important role in the initiation and progression of urological malignancies. In this review, we highlight the critical role of glyoxalases as regulators of tumorigenesis in the prostate through modulation of various critical signaling pathways, and provide an overview of the current knowledge on glyoxalases in bladder, kidney and testis cancers. We also discuss the promise and challenges for Glo1 inhibitors as future anti-prostate cancer (PCa) therapeutics and the potential of glyoxalases as biomarkers for PCa diagnosis.

## 1. Introduction

Urological cancers consist of prostate, kidney, bladder and testis cancers. Excluding testicular cancer, they are among the ten most frequent malignancies in man [1]. According to the most recent cancer statistics, renal, bladder and prostate cancers constituted more than 33% of all cancers in the United States in 2016 [2]. Despite improved primary prevention, early detection and treatment, urological cancers are still characterized by increasing incidence and mortality worldwide [1], and despite the significant progress in understanding the pathology of these malignancies, the study of the mechanisms involved remains an unmet, but essential, research goal if we are to define safer pharmacological therapies and prognostic factors, especially for the metastatic stage of these neoplastic diseases whose prognosis, unfortunately even today, remains poor. Prostate cancer (PCa) is a major health concern in the older male population all over the world and is the sixth most common cause of cancer-related deaths in the world [3]. Androgen receptor plays a crucial role in the development of PCa and androgen deprivation therapy is the first-line therapy for newly diagnosed PCa patients [4]. Nevertheless, most patients progress to castration-resistant PCa, very often associated with metastases [5]. Bladder cancer (BCa) incidence is three times higher among males than females [6]. Nearly 386,000 newly diagnosed cases and about 150,000 deaths are reported annually worldwide [4]. Renal cell carcinoma (RCC) is the eighth most common cancer in the United States [7] and its incidence is steadily rising in most areas of the world [8]. Total or partial nephrectomy is the optimal primary treatment. Nevertheless, RCC recurs in 20–40% of patients after resection, which is associated with a worse tumor stage and grade [9]. To further exacerbate the situation, at least some malignancies of the urinary tract are age-related, therefore, it is highly likely that their incidence may continue to rise as a result of global population aging.

Multiple factors are involved in the genesis and progression of urological malignancies, including hypoxia, hormone influence, genetic predisposition, oxidative and glycative stresses and inflammation [10,11,12,13,14,15,16,17,18]. With regards to oxidative stress, glycative stress and inflammation, a complex interplay is known to exist between these biological phenomena, so that each of them fuels the other in a fine-tuned regulatory circuitry.

**Figure 1:**
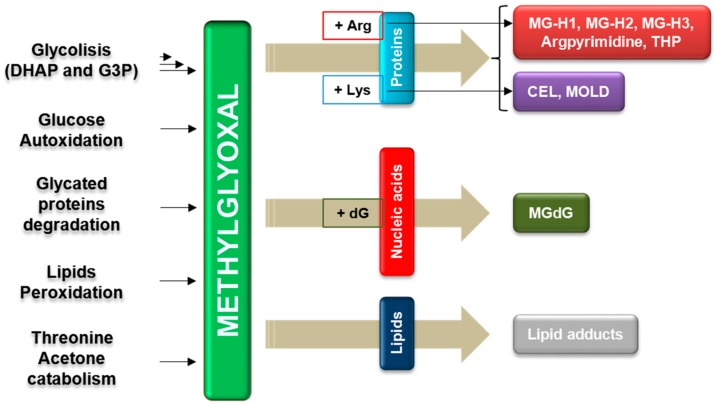
Formation and modification of macromolecules of/by methylglyoxal (MG). In cells, the majority of MG is formed non-enzymatically as a by-product of glycolysis, in particular, from the degradation of dihydroxyacetone phosphate (DHAP) and glyceraldehyde-3-phosphate (G3P) [22,23]. Minor sources of MG are derived from the hydrolysis and dephosphorylation of DHAP and G3P by triosephosphate isomerase [24], from aminoacetone during catabolism of threonine and from hydroxyacetone in the metabolism of acetone [25,26]. MG can also be formed by the decomposition of lipid peroxidation products [27,28], autoxidation of glucose and degradation of glycated proteins [29,30,31]. MG can modify arginine (Arg) residues of proteins to generate MG-derived hydroimidazolones (MG-H1, MG-H2, MG-H3), argpyrimidine and tetrahydropyrimidine (THP), as well as lysine (Lys) residues to form Nε-(1-carboxyethyl)lysine (CEL) and 1,3-di(Nε-lysino)-4-methyl-imidazolium (MOLD). MG also induces stable modifications of DNA bases (2′-deoxyguanosine, dG) and lipids.

A crucial common factor in this interplay is represented by methylglyoxal (MG), an endogenous dicarbonyl metabolite [19], formed during cell metabolism, especially glycolysis (Figure 1), so that MG is particularly abundant in actively proliferating cancerous cells, that primarily rely on glucose metabolism to support their elevated growth demands, according to the so-called “Warburg effect” [20]. MG is a potent glycating agent [19], able to rapidly and spontaneously react with free amino groups in proteins, with DNA and lipids, generating a large family of adducts, called advanced glycation end products (AGEs) [21] (Figure 1).

In particular, by reacting mainly with arginine residues of proteins, MG forms, primarily, hydroimidazolone adducts (MG-H1, MG-H2 and MG-H3) [32,33,34], and, secondly, argpyrimidine [35] and tetrahydropyrimidine (THP) [36]. MG can also react with lysine residues of proteins forming Nε-(1-carboxyethyl)lysine (CEL) [37] and 1,3-di(Nε-lysino)-4-methyl-imidazolium (MOLD) [38]. The interaction of MG with DNA leads preferentially to the formation of imidazopurinone MGdG [39,40,41,42], while, by reacting with basic phospholipids, MG forms lipid adducts [42,43]. MG is a crucial factor in the induction of oxidative stress and in the generation of reactive oxygen species (ROS) [40,44,45,46,47,48]. The major route by which MG generates ROS is by directly modifying proteins, e.g., mitochondrial membrane proteins [49,50], antioxidant enzymes [50,51], endothelial nitric oxide synthase [52] and NADPH oxidase [53]. Moreover, MG, mainly through MG-H1, which serves as a ligand for the receptor of AGEs (RAGE), is an important pro-inflammatory agent [40,54,55,56].

**Figure 2:**
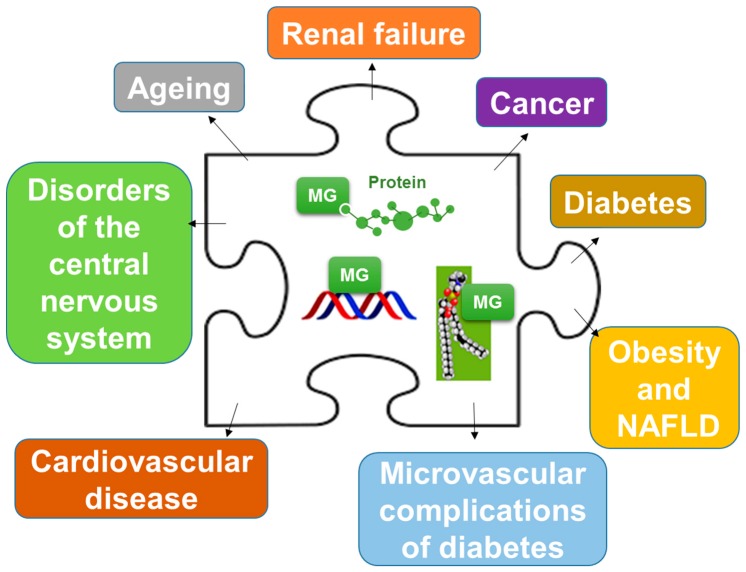
Involvement of methylglyoxal (MG)-derived advanced glycation end products (AGEs) in aging and disease. MG-derived AGEs are an important piece of the puzzle representing the pathogenesis of several health problems. NAFLD: non-alcoholic fatty liver disease.

Increased levels of MG and MG-modified macromolecules, particularly, MG-modified proteins (“the dicarbonyl proteome”, [41]) can drive several biological responses, including cell death [57,58,59,60,61,62], cell transformation [63,64,65,66] and senescence [67,68,69], contributing to cell and tissue dysfunction in aging and disease [41,70] (Figure 2).

Under physiological conditions, intracellular concentrations of MG and, consequently, of MG-derived AGEs, are strictly regulated. Methylglyoxal is metabolized mainly by glyoxalases [71,72,73,74,75,76,77], with minor metabolism by aldoketo reductases (AKRs) and aldehyde dehydrogenases (ADHs) [19,40,78,79,80,81] that convert it to hydroxyacetone and pyruvate, respectively [82]. Glyoxalases are cellular enzymes whose increased expression and activity in tumor tissues and/or cell lines [77,83,84,85], including those from the urogenital tract [86,87,88,89,90], have been widely documented [77,83,84,85,86,87,88,89,90]. In the present review, we provide an overview of the role of glyoxalases in the onset and progression of human urogenital malignancies and glyoxalases’ potential as targets for anticancer drug development and biomarkers for diagnosis or prognosis of urological cancers.

## 2. Glyoxalases

**Figure 3:**
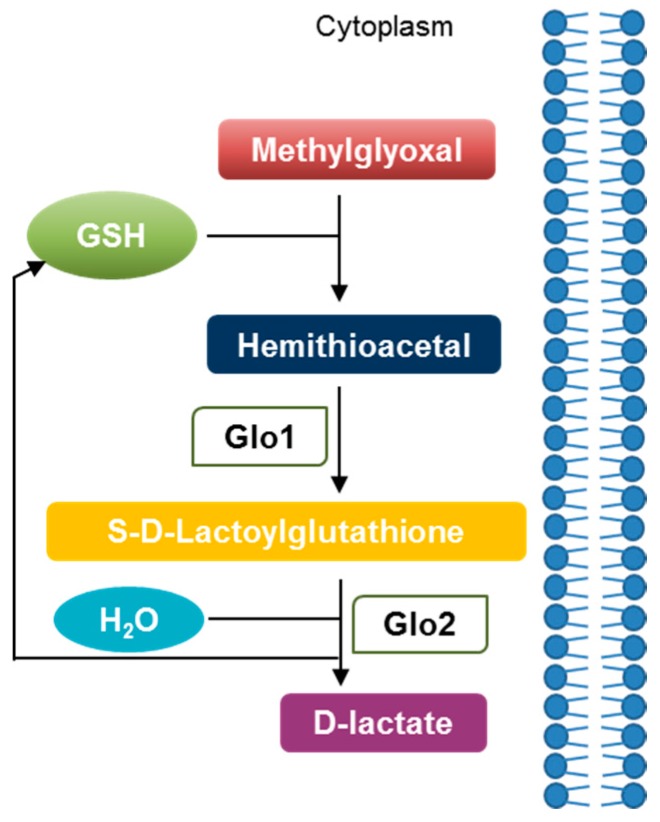
The glyoxalase system. The glyoxalase system consists of glyoxalase 1 (Glo1), glyoxalase 2 (Glo2), and a catalytic amount of glutathione (GSH). Acting as the rate-limiting enzyme, Glo1 transforms the hemithioacetal spontaneously formed between MG and GSH to the thioester S-d-lactoylglutathione. In turn, Glo2 catalyzes the hydrolysis of S-d-lactoylglutathione to d-lactate regenerating GSH.

Glyoxalases comprise two enzymes, named glyoxalase 1 (Glo1) and glyoxalase 2 (Glo2). The two enzymes have been historically described to work in tandem as part of a system where Glo1 catalyzes the isomerization of the hemithioacetal formed non-enzymatically by the reaction of MG with reduced glutathione (GSH) into S-d-lactoylglutathione, and Glo2 the hydrolysis of S-d-lactoylglutathione to d-lactate, thereby reforming GSH [21] (Figure 3).

**Figure 4:**
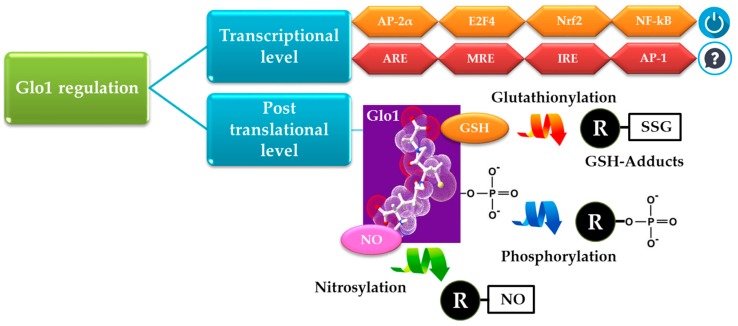
Mechanisms of glyoxalase 1 (Glo1) regulation. Glo1 can be regulated at transcriptional and post-translational levels. Transcriptional regulators are activator protein-2α (AP-2α), early gene 2 factor isoform 4 (E2F4), nuclear factor erythroid 2-related factor 2 (Nrf2), nuclear transcription factor–kB (NF-κB), antioxidant response (ARE), metal-response (MRE) and insulin-response (IRE) elements, and activator protein-1 (AP-1). It has been shown that AP-2α, E2F4, Nrf2 and NF-κB enhance (switch on symbol) the activity of Glo1 promoter, and up-regulate Glo1 expression. As to *Glo1* regulation by ARE, MRE, IRE and AP-1, a clear demonstration has not been provided yet (question mark symbol). Post-translational modification can occur via glutathionylation, phosphorylation and nitrosylation. GSH: reduced glutathione, NO: nitric oxide; R: the “variable” or “R” group of proteins.

Glo1 activity can be regulated through several mechanisms including transcriptional regulation [21,59,84,91] and by post-translational modifications [21]. In particular, the promoter region of *Glo1* contains various regulatory elements, including binding sites for activator protein-2α (AP-2α), early gene 2 factor isoform 4 (E2F4), nuclear transcription factor-κB (NF-κB), and activator protein-1 (AP-1), as well as antioxidant response (ARE), metal-response (MRE), and insulin-response (IRE) elements [91,92] (Figure 4). It has been shown that AP-2α, E2F4, nuclear factor erythroid 2-related factor 2 (Nrf2) and NF-κB enhance the activity of Glo1 promoter, and up-regulate Glo1 expression [93,94]. Phosphorylation, NO-mediated modification and glutathionylation have been described as post-translational modifications of Glo1 [21,95,96,97,98] (Figure 4).

**Figure 5:**
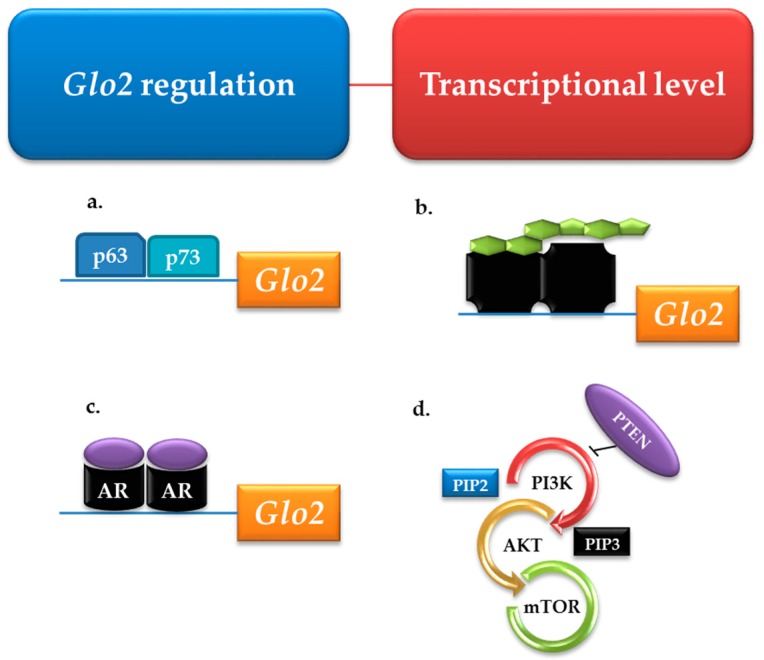
Transcriptional regulation of glyoxalase 2 (*Glo2*). Transcriptional positive regulators are (**a**) the transcription factors p63 and p73; (**b**) steroid hormones; (**c**) androgen receptor (AR) and (**d**) phosphoinositide 3-kinase (PI3K)/protein kinase B (AKT)/mammalian target of rapamycin (mTOR) signaling, one of the most important pathways involved in prostate cancer (PCa) progression, negatively regulated by the tumor suppressor phosphatase and tensin homologue (PTEN).

Glo2 expression can be up-regulated by the transcription factors p63 and p73 [99], steroid hormones [88], androgen receptor [90] and phosphatase and tensin homologue (PTEN)/ phosphoinositide 3-kinase (PI3K)/protein kinase B (AKT)/mammalian target of rapamycin (mTOR) signaling [89] (Figure 5). Extensive information about physical and chemical properties of glyoxalases has been largely described in excellent previous reviews [100,101,102,103,104].

## 3. Prostate Cancer (PCa)

### 3.1. Glyoxalase 1 (Glo1)

The first interest in the role of Glo1 in PCa dates back to the 1990s, when Ayoub et al. [105] and Di Ilio et al. [106] measured Glo1 specific activity in human tumor cell lines in vitro or cancerous and non-cancerous tissues. Although PCa tissues were poorly represented and PCa cell lines not clearly identified, these results suggested a role for Glo1 in PCa biology, its enzymatic activity being higher in cancerous cells than non-malignant ones. The first seminal observations of the potential role of Glo1 in PCa onset were made later by Davidson et al. [107] who performed a quantitative PAGE-based Glo1 assay on 22 PCa and ten normal prostate tissues to assess its activity, and thereby establish its clinical importance in this neoplasm. These results demonstrated that Glo1 activity differed substantially and significantly between non-cancerous and cancerous specimens, with a densitometric analysis showing differences in the range from five- to eight-fold in PCa compared with non-cancerous samples. Notably, a distinctly high activity of Glo1 was observed in all 22 PCa specimens and with little to no activity detected in ten out of ten non-malignant specimens. Although preliminary, these results provided new impetus to the investigation of Glo1 expression, either at mRNA, protein or functional level, in PCa tissues and normal counterparts or in differentially aggressive and invasive PCa [89,90,108,109,110]. Overall, these studies strongly suggested Glo1 as an important new target in PCa genesis and progression. Overexpression of Glo1 in PCa cells would maintain low intracellular levels of the glycolysis-derived cytotoxic metabolite MG, as a survival defense strategy. Indeed, in vitro studies performed with PCa cell lines, clearly demonstrated just such a role [57,58,89], in addition to pointing out the pro-survival function of this protein by eluding apoptosis in a mechanism involving the desensitization of NF-κB pathway [58]. Only one study, based on the proteomic analysis of normal and malignant prostate tissues, identified Glo1 among the proteins lost in PCa [111]. While this result was not further validated, it is worth raising a note of caution here. The role of Glo1 in PCa carcinogenesis has supported the view of Glo1 both as a marker for diagnosis of PCa and a potential target for the development of novel anti-cancer or chemotherapeutic agents for PCa, which were indeed confirmed by some studies. In particular, Chavan et al. [112] suggested serum Glo1 levels as a supplemental biomarker to differentiate between malignant and non-malignant diseases of the prostate in patients with PSA in the range of 4–20 ng/mL. Similarly, we first explored the association of Glo1 Ala111Glu (A111E) (419A/C) functional polymorphism, associated with a loss-of-function mutation. Our findings supported the idea of a pathogenic role of Glo1 in PCa as well as identified an additional candidate for risk assessment in PCa patients and an independent prognostic factor for survival [113]. Although further investigations with a larger number of participants are absolutely required to confirm these findings, these explorative studies have stimulated further investigations important for PCa diagnosis and encourage further progress in this field. So far, the gold standard in diagnosis for urological neoplasms involves tissue biopsy, and early screening methods are rare. Some existing biomarkers such as prostate-specific antigen (PSA) may be useful in PCa screening, but its utility is hampered by its poor specificity, leading to over-diagnosis and over-treatment, which limits its application [114,115,116]. With regards to Glo1 as a drug target, Sharkey et al. [117] provided the first demonstration that a competitive inhibitor of Glo1 effectively inhibited the growth of PCa tumors in mice, when delivered as the diethyl ester prodrug. Notably, the antitumor effects of this Glo1 inhibitor were comparable to those observed with the standard antitumor agents. Carmustine (BCNU) is an anticancer agent and another putative inhibitor of Glo1, while β-glucan is a unique, nontoxic polysaccharide extracted from mushrooms. In 2002, treatment of PC3 PCa cells with a combination of BCNU with β-glucan demonstrated a sensitized cytotoxic effect which was associated with a striking (approximately 80%) inactivation of Glo1 [118]. More recently, it has been also reported that curcumin inhibits Glo1 with the consequent intracellular accumulation of toxic levels of MG and GSH, that, by modulating various metabolic cellular pathways, inhibit growth of malignant cells [119]. Finally, Baunacke et al. [109] identified ethyl pyruvate as an agent targeting Glo1 that reduced some malignancy-associated properties of PCa cells while Valenti et al. [120] found that 3-bromopyruvate induced rapid human PCa cell death by affecting, among other targets, Glo1. Although these results convincingly suggested a crucial role for Glo1 in PCa, we believe that further clinical and/or in animal studies are necessary in the future before drawing definitive conclusions.

### 3.2. Glyoxalase 2 (Glo2)

In marked contrast to Glo1, the role of Glo2 in PCa has been relatively overlooked [89,90,105,106]. Ayoub et al. [105] and Di Ilio et al. [106] first evaluated Glo2 enzymatic activity in human PCa cell lines and tissues. They found that Glo2 enzyme activity in cancerous cells was not so different from that of non-cancerous cells. However, the small number of PCa samples considered in those studies significantly limited the strength of the conclusions drawn on the biological significance of Glo2 in this neoplasia. Only recently we demonstrated a causative role of Glo2 in PCa [89,90] providing in vivo and in vitro evidence for a role of this enzyme in prostate carcinogenesis. In particular, we showed that Glo2 was selectively expressed in PCa but not in the luminal compartment of the adjacent benign epithelium, consistently in all the examined cases (*n* = 20). Moreover, we demonstrated that Glo2 expression in malignant prostate cells was dependent on androgen receptor, in line with another previous exploratory study by our group [88], and was linked to enhanced cell proliferation and resistance to apoptosis through a mechanism involving the p53-p21 axis. Hence, our results demonstrated, for the first time, a role of Glo2 in prostate tumorigenesis as well as suggesting a possible mechanism. Both genetic and environmental factors participate in PCa pathogenesis [121,122]. Nevertheless, the molecular biology and mechanisms of prostate carcinogenesis remain to be further elucidated in order to identify additional diagnostic factors. As mentioned above, while the possibility of biomarkers for PCa has been investigated for some molecules, their prospects in clinical application still need to be further evaluated [114,115,116,123]. There is an urgent need for additional biomarkers that supplement PSA. Since we demonstrated that the luminal Glo2 is able to drive prostate tumorigenesis and that it is not expressed in benign luminal cells but only in PCa, we proposed that it might represent a novel marker in the pathological diagnosis of early PCa, by distinguishing between benign and malignant lesions. In fact, detection of Glo2 staining in luminal cells of the benign gland during diagnostic investigations might be indicative of an initial/ongoing neoplastic transformation process. In addition, we found in tissue sections, that Glo2 was also intensely expressed in the basal cells of benign glands, even though this basal cell-localized Glo2 was not involved in PCa genesis. The histological diagnosis of PCa, at least in difficult cases, is based upon prostate gland architectural and cytological features, with basal cell loss as a hallmark of malignancy. When the growth pattern is obscured, as can be the case in core needle biopsies with few suspicious glands, assessing these basal cells immunohistochemically is the usual auxiliary approach in confirming or excluding malignancy [124]. A variety of basal cell markers have been suggested. The standard markers used routinely in surgical pathology are high molecular weight cytokeratins (e.g., 34βE12, CK5/6) or p63, both of which stain basal cells with high sensitivity and specificity [124]. However, in some cases, false-negative staining may occur because of patchy cytoplasmic staining, making a definitive diagnosis difficult. We suggested that Glo2 immunostaining could represent a novel additional marker for basal cells in the benign prostate and its absence in malignant cells, consequent to the loss of basal cells, might have been useful as a complement to existing methods to facilitate the pathological diagnosis of PCa. In particular, since the loss of basal cells is an early event during tumorigenesis, we believe that Glo2 might be especially important for the early diagnosis of PCa. The concept of a metabolic pathway comprising Glo2 and Glo1, is a widely accepted idea. However, this view was first challenged in 1970 with a report of Glo2 deficiency in erythrocytes [125]. The authors studied this deficiency in family members for three generations demonstrating that this condition is transmitted as an autosomal recessive trait. Moreover, they observed that this deficiency was absent in leucocytes, while Glo1 activity was entirely normal in both leukocytes and erythrocytes, and was three times more active than Glo2 [126]. Additional studies in other eukaryotic organisms (reviewed in [101,126]) suggested further exceptions to the concept of a pathway composed of Glo1 and Glo2, acting sequentially. In our study, the results from IHC, confirmed in PCa cell models by multidisciplinary approaches, showed that the localization of Glo1 was opposite to that of Glo2. In all 20 cases of non-malignant glands, Glo1 staining was consistently observed only in the luminal but not in the basal cells, even with the use of different Glo1 antibody solutions, suggesting an independent role of the two proteins. Moreover, when attempting to demonstrate a possible role of Glo1 in association with Glo2 in PCa genesis via cell growth control we did not find any robust dependency. We suggest that Glo2 involvement in prostate tumorigenesis is independent of Glo1, at least as far as cell growth control is concerned, and/or that Glo1 may be involved in PCa genesis via the control of other tumorigenesis-related biological processes, possibly induced by factors in the cancer microenvironment. Further research is needed to clarify this intriguing finding. On the other hand, as mentioned above, we recently found that Glo2, this time in cooperation with Glo1, is also involved in human PCa progression as part of a mechanism driven by PTEN/PI3K/AKT/mTOR signaling with involvement of PKM2 and estrogen receptor α [89]. In particular, we found that both enzymes were up-regulated in both aggressive PCa tissues and cell lines and this up-regulation mediated maintenance of low intracellular levels of the glycolysis-derived cytotoxic metabolites MG and LSG, very likely as a survival defense strategy. Importantly, Glo2 silencing did not alter the behavior of benign cells, suggesting that targeting this protein might represent a strategy to selectively inhibit advanced PCa. Additional in vivo studies are absolutely required to further prove this function for both enzymes. Hence, despite significant research efforts and important new insights, the role of glyoxalases in the pathology and progression of PCa remains opaque; much more work must still be done.

## 4. Renal Cancer (RCa)

### 4.1. Glyoxalase 1 (Glo1)

Back in 1995, Di Ilio and colleagues evaluated Glo1 enzyme activity in the cytosolic fractions prepared from 15 samples of neoplastic and healthy kidney tissues from patients with primary renal cell carcinoma [106]. However, no significant differences were observed. Ten years later, we investigated Glo1 expression, at both mRNA and protein level, in a human renal carcinoma (clear cell adenocarcinoma, CCA), known to be strongly resistant to chemotherapeutic drugs, and in pair-matched normal tissues [87]. This study provided clear evidence for significantly higher (about nine-fold) levels of *Glo1* mRNA and activity (2.5-fold) and markedly reduced intracellular levels of pro-apoptotic MG in CCA than in control tissues, suggesting a mechanism explaining, at least in part, the refractoriness of CCA to a number of apoptosis-inducing chemotherapy treatments. In fact, despite the discovery of a number of new cytotoxic agents in the recent years, kidney cancer treatment is still unsatisfactory, mainly due to a marked resistance of this tumor towards these drugs. Hence, we suggested that the synthesis of new molecules selectively down-regulating Glo1 expression in cancer cells might be an alternative method of therapeutic treatment for this neoplasia, in particular CCA. Our study was later extended by Tanaka and colleagues who studied Glo1 expression in human renal tumor cell lines with diverse metastatic potential [127]. By using two-dimensional electrophoresis followed by liquid chromatography-tandem mass spectrometry, they found that protein Glo1 appeared to be directly proportional to the metastatic potential of the studied clones, and was also the only protein which consistently varied with the metastatic potentials of CCR clones: Glo1 was increased in highly metastatic cell lines compared with those with lower metastatic potential and this was confirmed by Western blot analysis. These results, in agreement with ours [87], suggested that the increased Glo1 expression in more aggressive CCR cells might provide advantages to the metastatic potential in aggressive clones, and that Glo1 inhibition might be a therapeutic approach for the treatment of metastatic CCR. A novel mechanism underlying increased Glo1 expression in tumors is *Glo1* gene amplification [72]. A quite recent study demonstrated a direct link between *Glo1* gene amplification, overexpression and increased sensitivity to Glo1 inhibition in 6.5% of RCa, and suggested that cell lines with *Glo1* amplification were significantly more sensitive to *Glo1* inhibition than those without. Moreover, it was suggested that Glo1 might represent a useful target for therapy in cancers with Glo1 amplification, such as RCa. As for studies on the association of the *Glo1* A111E (419A/C) polymorphism with the risk of RCa, they have been limited to only one study in a cohort of 214 patients with CCA [128]. This study demonstrated the link between the A111E Glo1 single nucleotide polymorphism (SNP) and the presence of CCR. In particular, a higher frequency of the C allele and genotype CC in patients with CCR was observed in comparison with the control group. This result would be in accordance with the fact that the C allele is linked to a higher enzymatic activity of Glo1, which was previously observed in another human urological malignancy [113]. However, although this study clearly demonstrated Glo1 involvement in kidney cancer, it identified some potential limitations, the most important of which was the lack of a biological link, since Glo1 activity was not measured either in tissue or blood samples.

### 4.2. Glyoxalase 2 (Glo2)

A clear connection between Glo2 and RCa was first established in 1995 when, once again, Di Ilio and colleagues [106] evaluated Glo2 enzyme activity in the cytosolic fractions from 15 samples of neoplastic or healthy kidney tissues of patients with primary RCC. In this study, Di Ilio et al. [106] revealed a significantly lower (approximately four-fold) Glo2 activity in malignant than in non-malignant kidney tissues. Only one study by our group followed this exploratory observation [87]. In agreement with Di Ilio et al. [106], we demonstrated a significant decrease of Glo2 enzyme activity (approximately two-fold) in the tumor compared with non-tumor tissues.

## 5. Bladder Cancer (BCa)

### Glyoxalase 1 (Glo1) and Gyoxalase 2 (Glo2)

Little information is available on glyoxalases in BCa [86,106]. A study [106] reported glyoxalases’ activity in six samples of normal bladder and in the tumor and non-tumor samples of eight bladders. Large inter-individual variations were observed in both Glo1 and Glo2 activities which, according to the authors, made the definition of normal baseline level difficult to set. Although no significant difference in Glo1 means activity was found between tumor and non-tumor samples of BCa, relatively higher tumor Glo1 activity, compared with the corresponding non-tumor tissues, was found in five samples. As to Glo2, no significant differences were observed between the cancerous component and the normal counterpart. Next, we studied *Glo1* and *Glo2* gene expression and enzyme activity in human BCa compared with the corresponding normal mucosa [86]. In particular, samples of these tissues were collected from 26 patients with superficial (SBCa) or invasive bladder cancer (IBCa). Both Glo1 mRNA expression and activity were significantly increased in SBCa samples, while they remained unchanged in IBCa samples, compared with the normal mucosa. Conversely, Glo2 showed a higher activity in the tumor (either SBCa or IBCa samples) versus normal tissues. These results suggested that the differing levels of Glo1 activity level and gene expression between the SBCa and IBCa samples might have helped in their differential diagnosis.

## 6. Testis Cancer (TCa)

### Glo1 and Glo2

As far as we know, only Di Ilio and colleagues reported on the activity of glyoxalases in TCa more than twenty years ago [106]. However, with the exception of one sample, they found Glo1 and Glo2 activity essentially identical between tumor and non-tumor samples. Significantly more research is needed to investigate the role of glyoxalases in this urogenital malignancy.

## 7. Concluding Remarks

### 7.1. Prostate Cancer (PCa)

Studies so far performed on the role of glyoxalases in urological cancers have mainly focused on PCa. These studies pointed to a role for Glo1 in prostate carcinogenesis, especially during PCa progression, according to which increased expression and activity of Glo1 would be permissive for survival and growth of tumors with relatively high glycolytic rates and related high fluxes of MG formation. Hence, in PCa, the use of specific inhibitors of Glo1 might indeed represent an effective anticancer strategy. Several natural compounds have been proposed as Glo1 inhibitors attenuating the growth of PCa [109,118,119,120]. In addition, these studies demonstrated that as well as Glo1, Glo2 represents a novel contributor to PCa progression [90] and, more importantly, that Glo1/Glo2 silencing did not alter the behavior of benign cells [90], suggesting that targeting glyoxalases may represent a strategy to selectively inhibit advanced PCa. There is an urgent need for in vivo studies to validate these important conclusions and move them towards potential clinical application. While the traditional cooperative role of Glo1/Glo2 appears valid in PCa progression, in PCa genesis it appears that Glo1 and Glo2 have independent roles, not working sequentially as a metabolic system, helping cast further doubt on whether the glyoxalase pathway is really a pathway [101], at least in some settings. Additional studies are needed to shed new light on the role of these intricate and intriguing proteins in the specific context of PCa and, more widely, in the oncologic ambit. Finally, studies performed on the role of glyoxalases in PCa demonstrated that Glo1 and Glo2 may represent novel additional markers in the pathological diagnosis of advanced and early PCa, respectively. We hope that urologist and oncologist alike, thanks to these new advances, may find renewed interest in these proteins which are frequently overlooked in cancer research.

### 7.2. Renal and Bladder Cancers (RCa, BCa)

The role of glyoxalases in renal and bladder cancers remains comparatively understudied. The little information that is available needs to be further corroborated but supports the potential use of these proteins as biomarkers of these neoplastic diseases, whose incidence continues to rapidly increase worldwide. In particular, glyoxalases might be useful in the early detection and post-surgical follow up [14,128] since no biomarkers currently exist for either application [129]. In fact, RCa and BCa lack specific predictive biomarkers meaning that diagnosis frequently only follows the presentation of advanced symptoms [129,130].

### 7.3. Testis Cancer (TCa)

The study of the role of glyoxalases in TCa is today basically still a “virgin field”. TCas are a group of heterogeneous, biologically diverse and clinically challenging neoplasms. Despite the relatively low incidence and mortality rates, a subgroup of patients with disseminated disease relapse after conventional therapy and have a dismal prognosis. Moreover, TCs afflict mostly young men and have therapeutic peculiarities, with some patients showing resistance to cisplatin-based treatments and others being troubled by irreversible side effects, such as infertility [131,132]. It is to be hoped that these disease features will provide powerful motivation for further research in this area.

## 8. Future Directions

Research on glyoxalases has experienced frequent and surprising turns ever since they were discovered. Research on glyoxalases in urological malignancies might reveal, as has already occurred in the past, novel and interesting roles of these proteins and bring them to the limelight once more.

## Abbreviations

Glo1	Glyoxalase 1
Glo2	Glyoxalase 2
MG	Methylglyoxal
AGEs	Advanced glycation end products
AKRs	Aldoketo reductases
ADHs	Aldehyde dehydrogenases
PCa	Prostate cancer
BCa	Bladder cancer
RCC	Renal cell carcinoma
RCa	Renal cancer
ROS	Reactive oxygen species
GSH	Glutathione
PSA	Prostate-specific antigen
BCNU	Carmustine
CCA	Clear cell adenocarcinoma
SNP	Single nucleotide polymorphism
SBCa	Superficial bladder cancer
IBCa	Invasive bladder cancer
TCa	Testis cancer
